# Analyses of optimal body mass index for infertile patients with either polycystic or non-polycystic ovary syndrome during assisted reproductive treatment in China

**DOI:** 10.1038/srep34538

**Published:** 2016-09-30

**Authors:** Fang Wang, Wei Dai, Xin-hong Yang, Yi-hong Guo, Ying-pu Sun

**Affiliations:** 1 Reproductive Medical Center, The First Affiliated Hospital of Zhengzhou University, Zhengzhou 450052, China

## Abstract

We observed the effect of body mass index (BMI) on pregnancy outcomes in Chinese patients undergoing assisted reproductive treatment (ART). All the patients were divided into polycystic ovary syndrome (PCOS) group and non-PCOS group, and then according to BMI, each group was subdivided into 6 subgroups: group 1 (BMI < 18 kg/m^2^), group 2 (18–20 kg/m^2^), group 3 (20–22 kg/m^2^), group 4 (22–24 kg/m^2^), group 5 (24–26 kg/m^2^) and group 6 (BMI > 26.0 kg/m^2^). We found that in 20 to 25-year-old patients, the pregnancy rate was not significantly correlated with BMI in PCOS patients; while in non-POCS patients, the pregnancy rate significantly decreased at the BMI cut-off point value of 24–26 kg/m^2^. The pregnancy rate significantly declined at the BMI cut-off point values of 22–24 kg/m^2^ and 18–20 kg/m^2^, respectively in 25 to 35-year-old and in over 35-year-old PCOS patients; while in over 25-year-old non-PCOS patients, no significant correlation between pregnancy rate and BMI was observed. We conclude that for under 25-year-old non-PCOS patients, ART should be performed after BMI is controlled under 26 kg/m^2^. For PCOS patients, if age is 25 to 35 years or over 35 years, BMI should be controlled below 24 kg/m^2^ or below 20 kg/m^2^, respectively.

World Health Organization (WHO) predicted that there would be about 230 million overweight adults and 700 million obese individuals in the world by 2015^1^. Obesity has become a global problem because it affects human reproductive health and increases the risks of diabetes mellitus, and cardiovascular and cerebrovascular diseases. Obesity may place pregnant women at the risks of abortion, gestational diabetes, gestational hypertension, premature birth and abdominal delivery. Moreover, the off-springs of obese pregnant women have high birth defect rate and are susceptible to adult-onset diabetes and obesity. Therefore the effect of pro-gestation body weights on pregnancy outcomes, especially on the assisted reproductive outcomes, increasingly arouses reproductive doctors’ attention.

Polycystic ovary syndrome (PCOS) is a kind of ovulation failure disease. PCOS clinical manifestations are different, but 35% to 65% of PCOS patients have overweight or obesity^2^. Therefore, the PCOS patients who receive assisted reproductive technology (ART) due to ovulation failure are usually observed as a special group.

Body mass index (BMI) is widely used to evaluate obesity. BMI is more reliable than either body height or weight in predicting pregnancy outcomes. WHO made that 25 kg/m^2^ < BMI ≤ 30 kg/m^2^ was regarded as overweight, and BMI >30 kg/m^2^ as obesity. However, BMI is generally lower in Chinese population than in western population, so China made an overweight reference standard (BMI > 24 kg/m^2^) for Chinese population^3^. At present, there still are some debates about the effect of BMI on outcomes of *in vitro* fertilization-embryo transfer (IVF-ET) or intra-cytoplasmic sperm injection (ICSI), due to differences in races, ovulation induction protocols, ART and statistical analyses. Moreover, little research has been done on the optimal BMI for pregnancy so far.

In this study, we observed the effect of BMI on early pregnancy outcomes of IVF/ICSI, providing an optimal BMI for ART.

## Materials and Methods

All study methods were approved by Institutional Review Board and Ethics Committee of the First Affiliated Hospital of Zhengzhou University, and were performed in accordance with relevant guidelines and regulations. The informed consent was obtained from all subjects.

### Subjects

Clinical data of 31155 infertile patients, who received IVF/ICSI for the first time in our center from January 2012 to December 2014, were retrospectively analyzed. Of the 31155 patients, 2632 were diagnosed with PCOS and 28523 with non-PCOS. Exclusion criteria: Patients had ① uterine malformation; ② endometriosis; ③ donor sperms or donor oocytes, or pre-implantation genetic diagnosis; ④ no oocytes due to poor ovarian response; ⑤ a repeat cycle; ⑥ a canceled cycle; ⑦ no embryos for embryo transfer; ⑧ hyperthyroidism and other endocrine-metabolic diseases.

Diagnostic criteria of PCOS raised by European Society for Human Reproduction and Embryology, and the American Society of Reproduction Medicine at Rotterdam meeting in 2003, were ① oligo-ovulation or anovulation; ② high testosterone (T) or clinical manifestations caused by high T such as hirsutism, acne and so on; ③ ultrasonography showing 12 small ovarian follicles with diameters of 2 to 9 mm in unilateral or bilateral ovaries, namely polycystic ovary; and/or increased ovarian size of more than 10 cm^3^ on each side on the 3^rd^ to 5^th^ day of menstruation or after progesterone with-drawl. Other conditions causing high T were excluded, such as hyperprolactinemia, thyroid diseases, congenital adrenal cortical hyperplasia, Cushing syndrome, androgen secretion tumors, atypical adrenal cortical hyperplasia of 21-hydroxylase deficiency, exogenous androgen administration and so on. The patients were diagnosed with PCOS when they had 2 of the 3 items above and other conditions causing high T were excluded^4^.

BMI = weight (kg)/height (m)^2^. According to the analyses of large scale data for Chinese population conducted by obese working team in Chinese Office of International Life Science Association, patients were divided into 6 groups, including group 1 (BMI < 18 kg/m^2^, n = 1134), group 2 (18–20 kg/m^2^, n = 5392), group 3 (20–22 kg/m^2^, n = 8037), group 4 (22–24 kg/m^2^, n = 7463), group 5 (24–26 kg/m^2^, n = 4644), and group 6 (BMI > 26 kg/m^2^, n = 4485). BMI in group 1 were regarded as low weight, BMI in group 2–4 as normal weight, BMI in group 5 as overweight, and BMI in group 6 as obesity.

### Ovarian stimulation scheme

Ovarian stimulation was performed with standard long protocol. After complete down-regulation [(FSH (follicle-stimulating hormone) < 5 IU/L, E_2_ (estradiol) < 50 pg/ml, LH (luteinizing hormone) < 5 IU/L, endometrium thickness <5 mm and diameter of the maximal follicle ≤5 mm)] using decapeptyl (Germany, gonadotropin releasing hormone agonist: GnRH-α) or diphereline (France), Gonal-F (Gn, FSH: follicle-stimulating hormone, Switzerland) was given. The initial dose was based on patients’ clinical features. Follicular development was monitored by transvaginal ultrasound. When the maximal follicle >14 mm, fasting blood was taken from every patient to determine the levels of LH, E_2_ and progesterone (P) every morning. Gn dose was adjusted based on follicle size and endocrine levels. When the maximal follicle was more than 20 mm and the follicles >16 mm accounted for more than 2/3 of total follicles, human chorionic gonadotrophin (hCG) was given. Thirty seven hours later, oocyte retrieval was performed under ultrasonic guidance followed by *in vitro* fertilization (IVF)/intracytoplasmic sperm injection (ICSI). Two to five days later, 1–3 embryos or one blastula was transferred. The numbers of fertilization, cleavage and high-quality embryos were recorded.

### Endocrine determination

When the maximal follicle >14 mm, 3 ml of fasting venous blood was taken from every patient every morning. The levels of LH, E_2_ and P were determined by electrochemiluminescence immunoassay. Intra-assay and inter-assay coefficients of variation (CV) all were <10%.

### Statistical analysis

Statistical treatment was performed with SAS9.1 software. Means and ratios were compared between groups using independent sample *t*-test, Chi-square test and Mantel-Haenszel test. Linear relation analysis between two variables was performed using Pearson correlation. The effect of each variable on the pregnancy rate was analyzed by binary logistic regression using size of test α = 0.05 (bilateral).

Variables of logistic analyses: In both PCOS patients and non-PCOS patients, BMI > 24 kg/m^2^ was regarded as overweight. According to BMI, there were 6 groups including <18 kg/m^2^, 18–20 kg/m^2^, 20–22 kg/m^2^, 22–24 kg/m^2^, 24–26 kg/m^2^ and >26 kg/m^2^. According to age, there were 3 groups including 20–25 years, 25–35 years and over 35 years. Statistical methods: The pregnancy rate-related variables including age, BMI, infertile duration, basal FSH, days of Gn administration, Gn dosage, number of retrieved oocytes, fertilization rate, cleavage rate, high-quality embryo rate, and number of transferred embryos first underwent univariate logistic regression. And then the variables selected by single factor logistic regression, were subjected to multivariate logistic regression. Their odds ratios (*OR*) and corresponding 95% confidence intervals (*CI*) were obtained.

### Ethical approval and informed consent

All study methods were approved by Institutional Review Board and Ethics Committee of the First Affiliated Hospital of Zhengzhou University. All the subjects enrolled into the study gave written informed consent to participate.

## Results

### General data

A total of 31155 patients were enrolled in this study. Of the 31155 patients, 2632 had PCOS and 28523 were non-PCOS. There were significant differences in age, BMI, infertile duration, basal LH level, basic T level, Gn dosage, days of Gn administration, number of retrieved oocytes, number of available embryos and clinical pregnancy rate between PCOS patients and non-PCOS patients. No significant differences were found in basal FSH level, number of transferred embryos, abortion rate and ectopic pregnancy rate between PCOS patients and non-PCOS patients (Table 1).

The proportion of overweight or obesity significantly increased with age in both PCOS patients and non-PCOS patients, and it was significantly higher in PCOS patients than in non-PCOS patients in any age ranges (Table 2).

### Logistic analyses of pregnancy rate-related variables

Univariate analyses showed that in PCOS patients, BMI, high-quality embryo ratio and number of transferred embryos were correlated with pregnancy rate; while in non-PCOS patients, age, BMI, infertile duration, basal FSH level, Gn dosage, fertilization rate, cleavage rate, high-quality embryo rate and number of transferred embryos were correlated with pregnancy rate (all *P* < 0.05). Multivariate analyses indicated that in both POCS patients (*OR* = 0.014, *95% CI* [0.777, 0.972]*, P* < 0.05) and non-PCOS patients (*OR* = 0.961, *95% CI* [0.938, 0.986]*, P* < 0.05) BMI was correlated with the pregnancy rate (Table 3).

### Relation between BMI and pregnancy rate

**Figure 1:**
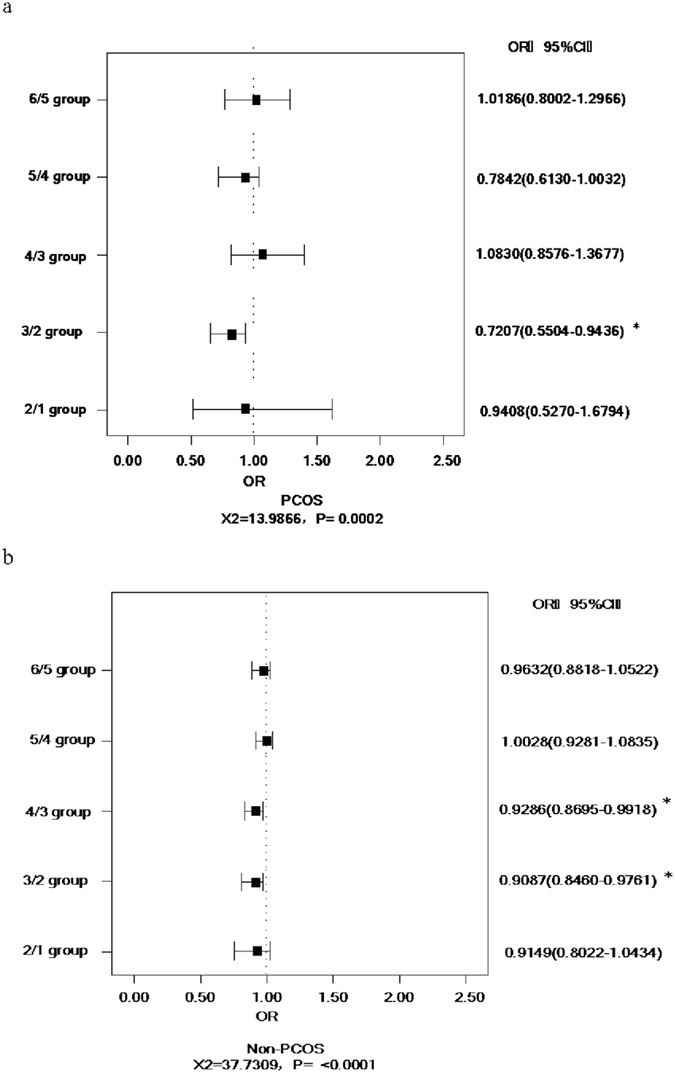
Relation between BMI and pregnancy rate in PCOS patients and non-PCOS patients. (**a**) in PCOS patients; (**b**) in non-PCOS patients. Notes: BMI: body mass index; PCOS: polycystic ovary syndrome; non-PCOS: non-polycystic ovary syndrome.

From Fig. 1a, we can see that the total pregnancy rate is higher in PCOS patients than in non-PCOS patients. With the increase in BMI, the pregnancy rate was on a downward trend. In PCOS patients, the pregnancy rate significantly decreased at the BMI cut-off point of 18–20 kg/m^2^ (*OR* = 0.720, 95% *CI* [0.5504, 0.9436], *P* < 0.05). From Fig. 1b, we can see that the pregnancy rate significantly decreases at the BMI cut-off points of 18–20 kg/m^2^ (*OR* = 0.9087, 95% *CI* [0.8460, 0.9761]) and 20–22 kg/m^2^ (*OR* = 0.9286, 95% *CI* [0.8695, 0.9918]) in non-PCOS patients.

### Relation between BMI and pregnancy rate in different age ranges

Both PCOS patients and non-PCOS patients were further stratified by age because age was one of the most important factors affecting pregnancy.

**Figure 2:**
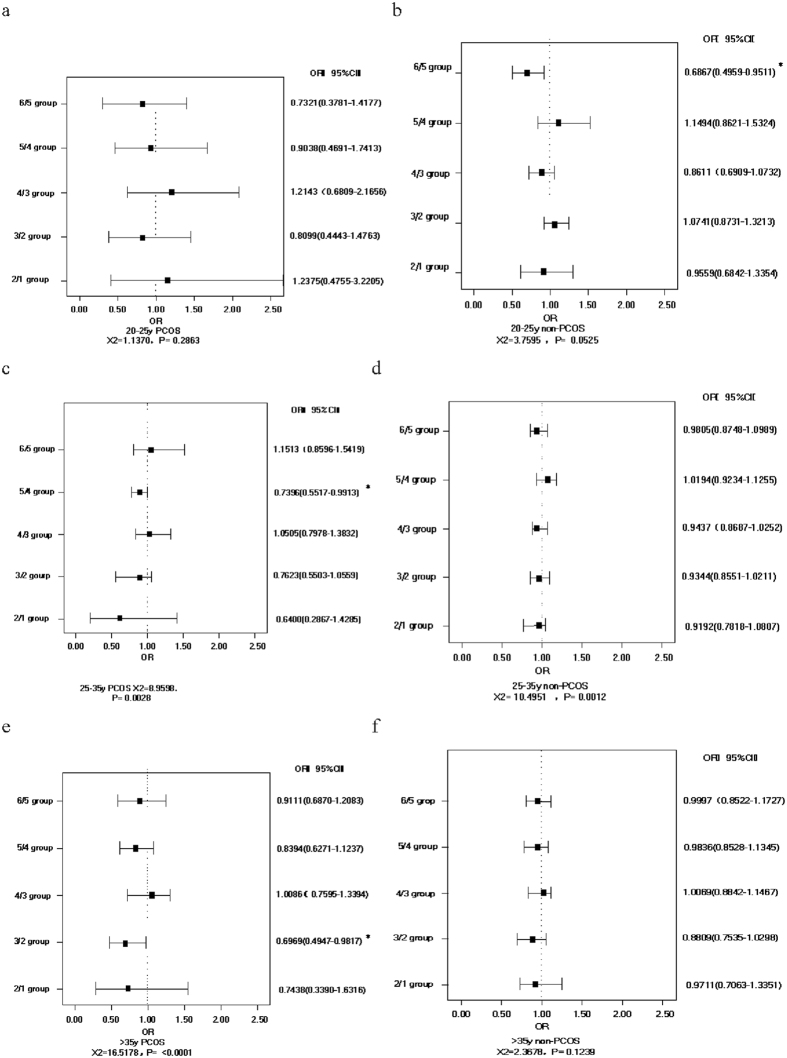
Relation between BMI and pregnancy rate. (**a**) in under 25-year-old PCOS patients; (**b**) in under 25-year-old non-PCOS patients; (**c**) in 25 to 35-year-old PCOS patients; (**d**) in 25 to 35-year-old non-PCOS patients; (**e**) in over 35-year-old PCOS patients; (**f**) in over 35-year-old non-PCOS patients. Notes: BMI: body mass index; PCOS: polycystic ovary syndrome; non-PCOS: non-polycystic ovary syndrome.

Figure 2a indicates that in under 25-year-old PCOS patients, although the pregnancy rate has descendent tendency as BMI increases, there are no significant cut-off points between BMI and pregnancy rate. From Fig. 2b, we can see that in under 25-year-old non-PCOS patients, the pregnancy rate declines as BMI increases, and it significantly decreases at the BMI cut-off point of 24–26 kg/m^2^ (*OR* = 0.6867, 95% *CI* [0.4959, 0.9511]).

Figure 2c indicates that in 25 to 35-year-old PCOS patients, the pregnancy rate significantly decreases at the BMI cut-off point of 22–24 kg/m^2^ (*OR* = 0.7396, 95% *CI* [0.5517, 0.9913]). From Fig. 2d, we can see that in 25 to 35-year-old non-PCOS patients, there are no significant cut-off points between BMI and the pregnancy rate.

Figure 2e displays that in over 35-year-old PCOS patients, the pregnancy rate significantly decreases at the BMI cut-off point of 18–20 kg/m^2^ (*OR* = 0.6969, 95% *CI* [0.4947,0.9817]). From Fig. 2f, we can see that in over 35-year-old non-PCOS patients, there are no significant cut-off points between BMI and the pregnancy rate.

### Relation between BMI and abortion rate

**Figure 3:**
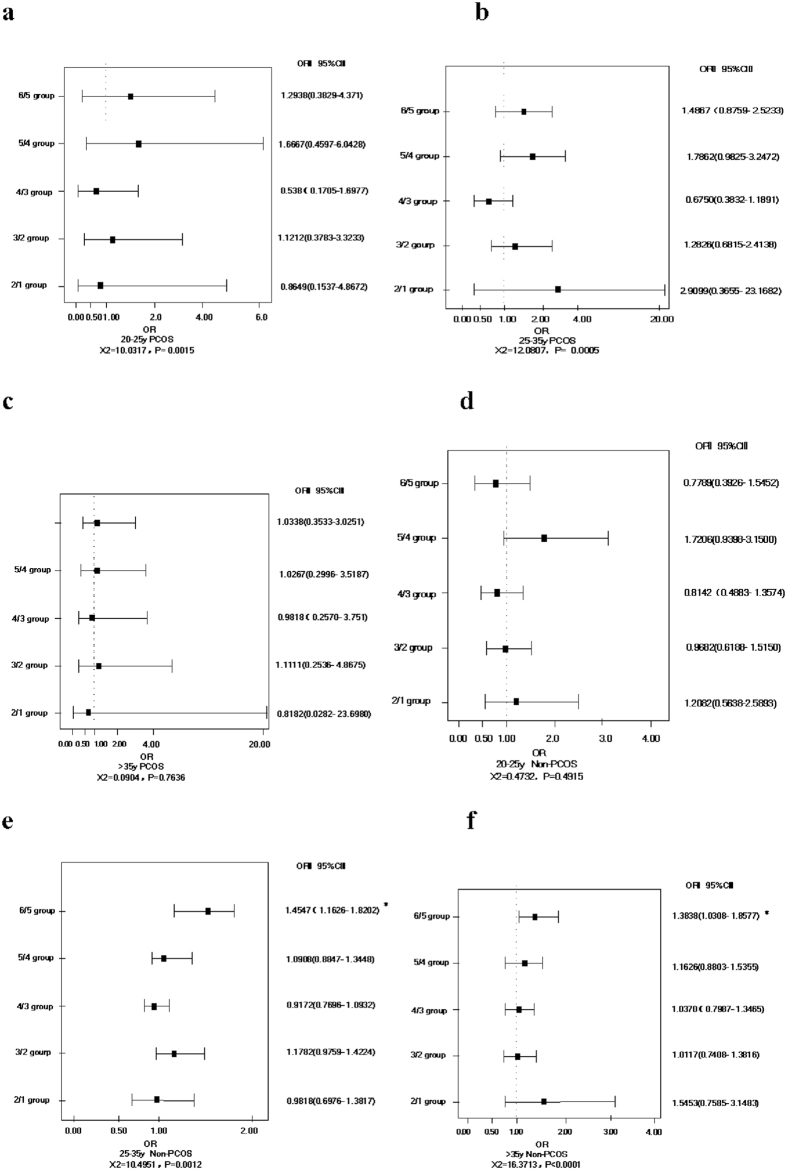
Relation between BMI and abortion rate in PCOS patients and non-PCOS patients in different age groups. (**a**) in under 25-year-old PCOS patients; (**b**) in under 25 to 35-year-old PCOS patients; (**c**) in over 35-year-old PCOS patients; (**d**) in under 25-year-old non-PCOS patients; (**e**) in under 25 to 35-year-old non-PCOS patients; (**f**) in over 35-year-old non-PCOS patients. Notes: BMI: body mass index; PCOS: polycystic ovary syndrome; non-PCOS: non-polycystic ovary syndrome.

From Fig. 3a–c, we can see that there are no significant correlations between BMI and abortion rate in PCOS patients in different age ranges. Figure 3d–f indicate that in under 25-year-old non-PCOS patients, abortion rate is not correlated with BMI; but in over 25-year-old non-PCOS patients, the abortion rate significantly increases at the BMI cut-off point of more than 26 kg/m^2^ (*OR* = 1.3838, 95% *CI* [1.0308,1.8577]).

## Discussion

To observe the effects of BMI on pregnancy rate, we retrospectively analyzed the clinical data from more than 30 thousand infertile patients who received IVF/ICSI, by the method of Mantel-Haenszel trend test. In clinical practice, obese patients are usually advised to reduce weight, but it has not yet been reported that how much weight loss is appropriate for age-varied PCOS patients or non-PCOS patients. This study aimed to explore these problems. We interestingly found that in under 25-year-old PCOS patients, BMI was not important for pregnancy rate, because there were not any cut-off points between BMI and the pregnancy rate although the pregnancy rate slightly declined as BMI increased. However, with the increase in age, the pregnancy rate significantly decreased at the BMI cut-off point of 22–24 kg/m^2^ in 25 to 35-year-old PCOS patients and at the BMI cut-off point of 18–20 kg/m^2^ in over 35-year-old PCOS patients. These findings provide a significant guidance in clinical practice for us. To obtain high pregnancy rate, for under 25-year-old PCOS patients, BMI does not require a stringent control; but for 25 to 35-year-old and over 35-year-old PCOS patients, the BMI should be controlled under 24 kg/m^2^ and 20 kg/m^2^, respectively. However, for over 35-year-old patients, more than half of them are obese and their BMI cut-off point is lower (20 kg/m^2^), so loss weight is arduous for them. The incidence of obesity is higher in PCOS patients than in normal population, which may be associated with genetic factor, and more likely long-term endocrine-metabolic disorder caused by PCOS.

To obtain high pregnancy rate, for under 25-year-old non-PCOS patients, the BMI should be controlled under 26 kg/m^2^; but for over 25-year-old non-PCOS patients, BMI does not require a stringent control. However, it is noteworthy that the abortion rate significantly increased as BMI was more than 26 kg/m^2^ in over 25-year-old non-PCOS patients.

### Relation between obesity and PCOS

In this study, compared with non-PCOS patients, PCOS patients were younger, and had higher BMI, shorter infertile duration, higher levels of basal LH and T, longer duration of Gn administration, lower Gn dosage, more numbers of retrieved oocytes and available embryos, and higher pregnancy rate; but there were no significant differences in abortion rate and ectopic pregnancy rate between non-PCOS patients and PCOS patients. During ovarian stimulation, PCOS patients readily have ovarian hyperstimulation syndrome (OHSS) manifesting more developmental follicles, more retrieved oocytes and high E_2_, so OHSS risk increases and cycle cancellation rate is high in PCOS patients. Therefore, in our reproductive center, for PCOS patient, the starting dosage of Gn (75–150 U/d) is low, avoiding OHSS as far as possible; if folliculogenesis is slow, Gn duration is usually prolonged or Gn dosage is increased when necessary. Moreover, most PCOS patients have high BMI, so Gn duration is likely also to be prolonged during ovarian stimulation.

BMI usually rises with age, especially in PCOS patients, because over half (55.65%) of PCOS women are obese. Obesity is associated with abnormal function of hypothalamic-pituitary-ovarian axis, and can affect the occurrence and progression of PCOS on multiple aspects. High BMI can affect both pathophysiology and clinical manifestations of POCS. In obese patients, insulin resistance and hyperinsulinemia can promote androgen secretion and can decrease the level of sex hormone-binding globulin, leading to hyperandrogenemia. In obese PCOS patients, hyperandrogenemia involves the dysfunction of hypothalamic-pituitary-ovarian axis. This leads to increased ratio of LH to FSH and relative FSH insufficiency, which, in a manner, allows follicular development to be arrested with anovulation and polycystic ovaries^5^,^6^. Therefore, obese PCOS patients are much more likely to have more remarked hyperandrogenemia and anovulation. Obesity and PCOS mutually affect each other^7^. There is even a report that obesity plays an essential role in the hypothalamic-pituitary-ovarian axis, because leptin replacement therapy has improved Gn secretion from the hypothalamus followed by occurrence of spontaneous development of follicles^8^. Moreover, obesity may increase release of some growth factors and inflammatory factors, which can allow ovaries to produce excessive androgen and can inhibit androgen aromatization into estrogen^9^.

### Effects of BMI on early pregnancy outcomes in PCOS patients

The total pregnancy rate was higher in PCOS patients than in non-PCOS patients without taking age into account, and it had descendent tendency as BMI increased. In the PCOS patients, the pregnancy rate significantly declined at the BMI cut-off point of 18–20 kg/m^2^. With taking age into account, in under 25-year-old PCOS patients, BMI was not significantly correlated with early pregnancy outcomes because there was no a significant cut-off point between the pregnancy rate and BMI, although the pregnancy rate had a downward trend as BMI increased. This may be that young patients generally have high-quality oocytes and embryos, which partly offset the negative effect of high BMI on pregnancy rate. With the increase in age, the pregnancy rate significantly decreased at the BMI cut-off point of 22–24 kg/m^2^ in 25 to 35-year-old PCOS patients and at the BMI cut-off point of 18–20 kg/m^2^ in over 35-year-old PCOS patients. In obese PCOS patients, hyperinsulinism can affect the development of oocyte and embryo, leading to reduction of fertilization rate, embryo implantation rate, clinical pregnancy rate and live birth rate^10^. Loss weight is an approach of successful ART for obese PCOS patients. BMI should be controlled under 24 kg/m^2^ in 25 to 35-year-old PCOS patient and under 20 kg/m^2^ in over 35-year-old PCOS patients, respectively. Thin PCOS patients are more conducive to ART outcomes than obese PCOS patients^11^.

### Effect of BMI on early pregnancy outcomes in non-PCOS patients

Without taking age into account, the pregnancy rate significantly declined at the BMI cut-off points of 18–20 kg/m^2^ and 20–22 kg/m^2^, respectively, in non-PCOS patients. With taking age into account, in order to obtain a better pregnancy rate, the ideal BMI was below 26 kg/m^2^ in the under 25-year-old non-PCOS patients; while in the over 25-year-old non-PCOS patients, BMI was not significantly correlated with the early pregnancy outcomes. At present, there has been considerable debate about the effect of high BMI on post-ART pregnancy outcomes. This may be related to different study populations, diagnostic criteria for BMI, ovarian stimulation protocols, drug species and so on. It has been reported that high BMI can decrease IVF/ICSI embryo implantation rate, clinical pregnancy rate and early abortion rate^12^. However, it has also been described that high BMI can decrease ovarian response, but it fails to affect IVF/ICSI early pregnancy outcomes^13^. A retrospective study has indicated that in 38- and under 38-year-old non-PCOS patients, BMI is not significant correlated with embryo quality, but the pregnancy rate is significantly decreased when BMI is more than 40 kg/m^2 ^14. A meta-analysis has displayed that amongst different pregestational BMI groups, there are no significant differences in clinical pregnancy rate and abortion rate, and further there also are no significant differences in live birth rate and singleton live birth rate, suggesting that BMI fails to affect IVF/ICSI live birth rate and singleton live birth rate^15^. Our study showed that in over 25-year-old non-PCOS patients, BMI was not significantly correlated with the early pregnancy outcomes, suggesting that under individualized ovarian stimulation strategy, BMI might not affect ART early pregnancy outcomes of non-PCOS patients.

### Relation between BMI and abortion

This study indicated no significant correlation between BMI and abortion rate in PCOS patients. This result is not consistent with other report^16^. It is generally accepted that PCOS patients readily have inadequate luteal function which is associated with abortion. However, in this study, all the patients had received luteal support during IVF/ICSI, so the abortion rate was not markedly changed. In under 25-year-old non-PCOS patients, the abortion rate was not significantly correlated with BMI; but in over 25-year-old non-PCOS patients, the abortion significantly decreased when BMI was more than 26 kg/m^2^. For non-PCOS patients, there are many factors to affect pregnancy outcomes. A meta-analysis of 36 original studies conducted from 1966 to 2010 and including a total of 47967 patients who received IVF/ICSI, indicated that the clinical pregnancy rate and live birth rate were significantly lower, and the abortion rate was relatively higher in overweight or obese pregnant women (BMI ≥ 25 kg/m^2^) than in normal-weight ones (BMI < 25 kg/m^2^)^17^. It has not been clear that how BMI affect the pregnancy outcomes. This may be that ① high BMI is likely to affect the quality of gametes and embryos, and then further affects the pregnancy outcomes of IVF/ICSI^18^; and ② high BMI alters endometrial environment^19^. Hormone levels are closely associated with adipose tissue volume in bodies. In obese women, excessively thick endometrium caused by high estrogen, is unfavourable for ART-successful pregnancy. Moreover, endometrial polyps may affect the environment of uterine cavity, leading to poor pregnancy rate. Onalan *et al.*^20^ have reported that obesity is related to both the occurrence and the prognosis of endometrial polyps. Therefore, obesity increases the abortion rate probably by altering intra-uterine environment.

### Advantages and limitations in this study

In this study, the results are relatively reliable, because all patients received standard long protocol, sample size was large and grouping was detailed. However, this study is retrospective, so some factors may affect pregnancy outcomes. For example, increasing BMI is correlated with increasing dose of stimulation^21^. This may result in the deviation of statistical results. We will try to carry out a rigorous control study to observe whether interventions in overweight or obesity improve pregnancy rate.

## Conclusion

For PCOS patients, if age is under 25 years, there are no strict requirements on BMI; if age is between 25 and 35 years, the BMI should be controlled under 24 kg/m^2^; if age is over 35 years, the BMI even should be controlled under 20 kg/m^2^. For non-PCOS patients, if age is under 25 years, the BMI should be controlled under 26 kg/m^2^ to obtain better pregnancy rate; if age is over 25 years, the BMI also should be controlled under 26 kg/m^2^ to reduce abortion rate.

## Additional Information

**How to cite this article**: Wang, F. *et al.* Analyses of optimal body mass index for infertile patients with either polycystic or non-polycystic ovary syndrome during assisted reproductive treatment in China. *Sci. Rep.* **6**, 34538; doi: 10.1038/srep34538 (2016).

## Figures and Tables

**Table 1:** Features of PCOS patients and non-PCOS patients undergoing their first IVF/ICSI cycles.

Parameter	n = 28523 (non-PCOS)	n = 2632 (PCOS)	*P*
Age (year)	32.56 ± 7.71	30.27 ± 4.92	0.000
BMI (kg/m^2^)	22.72 ± 3.12	23.60 ± 3.50	0.029
Infertile duration (year)	4.20 ± 3.43	4.03 ± 2.70	0.000
Basal FSH (IU/L)	8.08 ± 2.01	6.16 ± 1.49	0.189
Basal LH (IU/L)	6.18 ± 6.17	9.22 ± 7.60	0.000
Basal T (ng/ml)	0.82 ± 4.93	1.24 ± 6.82	0.002
IVF	19163	1596	
ICSI	9030	934	
primary infertility	17193 (60.28%)	2097 (79.67%)	0.000
secondary infertility	11330 (39.72%)	535 (20.33%)	0.000
Gn (days)	9.40 ± 6.34	10.07 ± 8.33	0.000
Gn dosage (IU)	1844.9 ± 1203.13	899.6 ± 903.45	0.000
No. of retrieved oocytes	10.75 ± 5.76	14.11 ± 5.93	0.000
No. of available embryos	5.41 ± 3.33	7.21 ± 4.41	0.000
No. of transferred embryos	2.06 ± 0.59	2.06 ± 0.59	0.908
Clinical pregnancy rate (%)	45.30% (12920/28523)	47.87% (1260/2632)	0.011
Abortion rate (%)	7.29% (1160/15921)	6.83% (93/1361)	0.536
Ectopic pregnancy rate (%)	2.09% (332/15921)	2.79% (38/1361)	0.084

Notes: PCOS: polycystic ovary syndrome; non-PCOS: non-polycystic ovary syndrome; IVF: *in vitro* fertilization; ICSI: intracytoplasmic sperm injection; BMI: body mass index; FSH: follicular stimulation hormone; LH: luteinizing hormone; T: testosterone; Gn: gonadotropin.

**Table 2:** The proportion of overweight or obesity in both PCOS patients and non-PCOS patients (%).

Age (year)	PCOS (BMI > 24 kg/m^2^)	Non-PCOS (BMI > 24 kg/m^2^)	*P*
20–25	34.46% (153/444)	18.77% (515/2744)	0.000
26–35	40.85% (739/1809)	26.68% (4748/17793)	0.000
>35	55.65% (207/372)	33.31% (2663/7993)	0.000
*P*	0.000	0.000	

Notes: PCOS: polycystic ovary syndrome; non-PCOS: non-polycystic ovary syndrome; BMI: body mass index.

**Table 3:** Logistic analyses of pregnancy rate-related variables in PCOS and non-PCOS patients.

	PCOS		non-PCOS	
	COR (95% CI)	AOR (95% CI)	COR (95% CI)	AOR (95% CI)
Age (year)	0.989 (0.963–1.106)	0.993 (0.968–1.032)	0.948 (0.943–0.953)*	0.968 (0.961–1.975)*
BMI (Kg/m^2^)	0.866 (0.787–0.953)*	0.014 (0.777–0.972)*	0.93 (0.91–0.951)*	0.961 (0.938–0.986)*
Infertile duration (year)	0.96 (0.912–1.011)	0.303 (0.914–1.028)	0.943 (0.935–0.952)*	0.969 (0.959–0.979)*
Basal FSH (IU/L)	1.019 (0.935–1.111)	0.646 (0.885–1.078)	0.977 (0.966–0.988)*	0.999 (0.996–1.003)
Basal LH (IU/L)	0.999 (0.983–1.015)	0.912 (0.982–1.017)	1.001 (0.998–1.004)	0.996 (0.99–1.002)
Basal T (ng/mL)	0.998 (0.98–1.015)	0.886 (0.981–1.017)	1.005 (0.998–1.012)	1.002 (0.994–1.01)
Gn duration (day)	1.035 (0.975–1.099)	0.663 (0.919–1.141)	0.988 (0.974–1.002)	1.051 (1.026–1.076) *
Gn dosage (IU)	1 (1–1)	1 (1–1)	1 (1–1)*	1 (1–1)*
No. of retrieved oocytes	0.989 (0.967–1.011)	0.785 (0.977–1.031)	1.027 (1.021–1.032)	1.557 (1.322–1.832)*
Fertilization rate (%)	1.22 (0.659–2.259)	0.209 (0.767–3.367)	1.406 (1.23–1.607)*	1.02 (1.014–1.027)*
Cleavage rate (%)	4.263 (0.422–43.083)	0.605 (0.16–23.163)	1.933 (1.351–2.767)*	1.975 (1.303–2.995)*
High quality embryo rate (%)	2.148 (1.26–3.662)*	0.022 (1.102–3.522)*	1.956 (1.744–2.194)*	2.284 (2.008–2.598)*
No of transferred embryos	1.553 (1.181–2.042)*	0.009 (1.113–2.116)*	1.474 (1.389–1.564)*	1.652 (1.545–1.767)*

*Indicates *P* < 0.05. PCOS: polycystic ovary syndrome; non-PCOS: non-polycystic ovary syndrome; BMI: body mass index; FSH: follicular stimulation hormone; LH: luteinizing hormone; T: testosterone; Gn: gonadotropin; COR: crude) odds ratio; AOR: adjusted odds ratio; CI, confidence interval. *P < 0.05.
